# Supplementary material for: Analysis of Attitudes and Practices towards the Influenza Vaccine in High-Risk Adults in Poland
Source: Vaccines (Basel). 2024 Mar 21;12(3):341. doi: 10.3390/vaccines12030341 (PMC10975961; doi:10.3390/vaccines12030341)
Supplement: Supplementary file 1 [file vaccines-12-00341-s001.zip › vaccines-2885580-supplementary.pdf]

QUESTIONNAIRE

1. Consent to participate in the survey

a. Yes
b. No

2. Where did you obtain the link to the study?

A. From e-mail
b. From Facebook

3. Gender

a. Female

b. Male

4. Age (years)

a. ≤30

b. 31-40

c. 41-50

d. >50

5. Education

a. Elementary

b. Middle school

c. Vocational school

d. High school

e. College/University

6. Place of residence

a. Village

b. Town with up to 50,000 residents

c. Town with up to 100,000 residents

d. Town with up to 250,000 residents

e. City with over 250,000 residents

7. Are you of indigenous ancestry (e.g., Kashubian)?

a. yes

b. no

8. How do you understand the phrase "40-70% vaccine effectiveness against the flu"?

a. 40-70% of vaccinated people will be immune to the flu

b. A vaccinated person will have a 40-70% chance of getting the flu

c. If a person had a 40-70% chance of getting the flu before vaccination, they now have half of that chance

d. I don't know

9. Do you have any of the following chronic diseases?

a. Asthma

b. BMI > 40 kg/m

c. Cancer

d. Chronic CSF leak

e. Chronic lung disease

f. Chronic kidney disease

g. Chronic liver disease

h. Hypertension

i. Heart disease

j. Respiratory secretion impairment

k. Immune disorder or immune suppression

l. Diabetes or other metabolic diseases

ł. Spleen problems or removal

10. Are you pregnant?

a. yes

b. no

11. Are you a long-term care resident?

a. yes

b. no

12. Do you know what the risk groups for influenza infection are?

a. yes

b. no

13. Have you been confirmed to have a SARS-CoV-2 infection in the last season (2022-2023)?

a. yes

b. no

14. Have you been confirmed to have a flu virus infection in the last season(2022-2023)?

a. yes

b. no

15. Have you ever received the flu vaccine?

a. yes

b. no

16. Did you experience any adverse effects from receiving the flu vaccine?

a. yes

b. no

17. How severe were the adverse effects you experienced after receiving the flu vaccine?

a. mild

b. moderate

c. severe

18. What adverse effects occurred after receiving the flu vaccine? (You can select multiple answers)

   a. Runny or stuffy nose

   b. Fatigue

   c. Headache

d. Muscle pain

e. Chills

f. Pain in the arms, legs, or other joints

g. Swelling at the injection site

h. Fever

i. Nausea/vomiting

j. Swelling/tenderness in the armpit

k. Redness at the injection site

l. Reduced appetite

m. Sleep problems

n. Nosebleed

o. Abdominal pain

p. Rash

q. Shortness of breath

r. Chest pain

19. Did you receive both: the flu and SARS-CoV-2 vaccines during the 2022-2023 season?

a. yes

b. no

20. How many doses of the SARS-CoV-2 vaccine have you received during the season 2022-2023?

a. 0

b. 1

c. 2

d. 3

e. 4

21. In your opinion, does the flu vaccine you've received alleviate the symptoms of a SARS-CoV-2 infection?

a. yes

b. no

22. Do you plan to get regular flu vaccinations in the future seasons?

a. yes.

b. no

23. What primarily motivates you to get flu vaccinations?

a. General promotion of vaccinations due to the emergence of SARS-CoV-2

b. Alleviating flu symptoms

c. Social responsibility

d. Alleviating symptoms associated with infections by other viruses, including SARS-CoV-2

e. I or my close ones are at risk of flu infection

f. I or my close ones frequently get the flu

g. I didn't receive a flu vaccine

h. Other (please specify)

24. Among the following, please select one answer that you believe is most important when getting flu vaccinations:

a. Flu vaccination can help differentiate other viral diseases, including SARS-Cov-2

b. By getting the flu vaccine, we protect at-risk individuals, including the elderly and those with diabetes, lung diseases, heart failure, and other chronic illnesses

c. Getting the flu vaccine contributes to improving the healthcare system

d. Increasing herd immunity

e. None

25. Please state the reason why you have not received flu vaccinations:

a. I don't have time

b. Fear of adverse events

c. I believe vaccines are not effective in protection

d. Organizational reasons beyond my control (e.g., lack of vaccines, long queues)

e. Not applicable (I got the flu vaccine)
